# Nucleotide Analogue-Related Proximal Renal Tubular Dysfunction during Long-Term Treatment of Chronic Hepatitis B: A Cross-Sectional Study

**DOI:** 10.1155/2016/2952635

**Published:** 2016-10-31

**Authors:** Abhasnee Sobhonslidsuk, Jirachaya Wanichanuwat, Pawin Numthavaj, Areepan Sophonsritsuk, Supanna Petraksa, Alongkorn Pugasub, Paisan Jittorntam, Anucha Kongsomgan, Sittiruk Roytrakul, Bunyong Phakdeekitcharoen

**Affiliations:** ^1^Division of Gastroenterology and Hepatology, Department of Medicine, Faculty of Medicine, Ramathibodi Hospital, Mahidol University, Bangkok 10400, Thailand; ^2^Section for Clinical Epidemiology and Biostatistics, Faculty of Medicine, Ramathibodi Hospital, Mahidol University, Bangkok 10400, Thailand; ^3^Department of Obstetrics and Gynecology, Faculty of Medicine, Ramathibodi Hospital, Mahidol University, Bangkok 10400, Thailand; ^4^Office of Research Academic and Innovation, Faculty of Medicine, Ramathibodi Hospital, Mahidol University, Bangkok 10400, Thailand; ^5^Department of Pathology, Faculty of Medicine, Ramathibodi Hospital, Mahidol University, Bangkok 10400, Thailand; ^6^National Center for Genetic Engineering and Biotechnology, Pathumthani 12120, Thailand; ^7^Division of Nephrology, Department of Medicine, Faculty of Medicine, Ramathibodi Hospital, Mahidol University, Bangkok 10400, Thailand

## Abstract

*Background*. There have been few reports of nucleotide analogue-related renal tubular dysfunction (RTD) in CHB patients. We assessed the prevalence and presentation of nucleotide analogue-related proximal RTD.* Methods*. A cross-sectional study was performed in CHB patients taking nucleotide analogues. Inclusion criteria were patients who were on adefovir or tenofovir as mono- or add-on therapy with lamivudine (LAM) >1 year. Serum and urine were collected. Fractional excretion of phosphate (FEPO_4_), uric acid (FEUA), and potassium was calculated. Renal losses were defined based on the criteria: protein (24-hour urine protein >150 mg), glucose (glycosuria with normoglycemia), phosphate (FEPO_4_ >18%), uric acid (FEUA >15%), potassium (renal potassium losses with hypokalemia), and bicarbonate (normal gap acidosis). Subclinical and overt proximal RTD were defined when 2 and ≥3 criteria presented.* Results*. Ninety-two patients were enrolled. The mean duration of nucleotide analogue taking was 55.1 ± 29.6 months. Proximal RTD was found in 24 (26.1%) patients (subclinical 15 (16.3%) and overt 9 (9.8%)). The severity of RTD was associated with the duration of nucleotide analogue (*P* = 0.01).* Conclusions*. The prevalence of proximal RTD in CHB patients taking nucleotide analogues was 26%. The severity of RTD was associated with the treatment duration. Comprehensive testing is necessary for early detecting nucleotide analogue-related nephrotoxicity.

## 1. Introduction

Over 350 million people around the world have been diagnosed with chronic hepatitis B (CHB) [1]. Progression of hepatitis B virus (HBV) related chronic liver diseases depends on host and viral factors [1]. Risks of cirrhosis and hepatocellular carcinoma development increase significantly along with increase in baseline HBV viral load [2, 3]. In patients infected with HBV, long-term treatment with antiviral drugs such as lamivudine (LAM) slows disease progression by reducing the risk and incidence of hepatic decompensation and hepatocellular carcinoma until HBV resistant strains emerge [4]. Nucleoside and nucleotide analogues with a strong potency and high genetic barrier such as entecavir (ETV) and tenofovir disoproxil fumarate (TDF) are recommended as the first-line therapy of CHB [5–7]. When drug resistant HBV develops or incomplete virological response occurs, following the use of a low genetic barrier nucleoside analogue (e.g., LAM), a switch from LAM to TDF, or adding adefovir dipivoxil (ADV) or TDF to LAM is recommended [5, 8, 9]. ADV, a nucleotide analogue of adenosine monophosphate, was approved for CHB treatment in 2002 [8, 9]. It was followed by an approval of TDF in naïve and LAM resistant CHB in 2008 [5, 6, 9]. TDF is structurally similar to ADV but has a more potent antiviral effect on HBV [9, 10].

A dose of 30 mg daily of ADV has been shown in a randomized trial to be associated with mild renal dysfunction and hypophosphatemia, whereas a 10 mg daily dose has been shown to carry no such risk [11]; therefore, the 10 mg dose has been chosen to be the recommended daily dose of ADV in CHB treatment. In clinical practice, TDF has been believed to be safer in terms of nephrotoxicity than ADV [12], and this has been supported by previous studies which showed that TDF was associated with a low incidence of renal dysfunction [13–15]. On the other hand, subsequent small studies on the safety of ADV and TDF have shown that long-term treatment with ADV or TDF could potentially cause renal dysfunction, hypophosphatemia, and impaired renal tubular phosphate reabsorption [16–19]. Long-term ADV and TDF treatment were both associated with proximal RTD in 15% of patients [19]. The inadequacies of these studies were small sample size, insufficient assessment of proximal renal tubular function, or retrospective conducted studies [15–19]. Due to the limitation of previous reports in term of low-quality of data and the short duration of follow-up, the recent guideline of the American Association for the Study of Liver Diseases (AASLD) recommends no preference between ETV and TDF regarding possible long-term risks of renal and bone complications [7]. The available literatures are casting doubt on the long-term safety of nucleotide analogue treatment in CHB patients.

In order to evaluate the possible risk of nucleotide analogues, we systematically assessed the prevalence, clinical presentation, and risk factors of nucleotide analogue-related RTD in CHB patients, who were treated with long-term nucleotide analogues.

## 2. Materials and Methods

### 2.1. Patients

A cross-sectional study was conducted at the liver clinics of Ramathibodi Hospital, a tertiary care center, between 1 June 2014 and 31 March 2015. The study was approved by the Committee on Human Rights related to Research Involving Human Subjects, Faculty of Medicine, Ramathibodi Hospital, Mahidol University. The study was performed according to the 1975 Declaration of Helsinki. Informed consent was obtained from the subjects before enrollment. CHB patients with age over 18 years, having received nucleotide analogues (e.g., ADV and TDF) as mono- or add-on therapy for more than 1 year and having serum creatinine level less than 1.5 mg/dL, were enrolled consecutively. Exclusion criteria included pregnancy, decompensated cirrhosis, coinfected with human immune deficiency virus or hepatitis C virus, and the presence of glomerular or tubulointerstitial diseases secondary to poorly controlled conditions such as type 2 diabetes, hypertension. Clinical information was obtained from medical records and the hospital database.

### 2.2. Sample Collection and Laboratory Testing

Fasting serum, spot, and 24-hour urine samples were collected after informed consent was obtained. Fasting serum samples were assayed for liver function test, glucose, blood urea nitrogen (BUN), creatinine, electrolyte, phosphate, and uric acid. Twenty-four-hour urine was tested for protein, creatinine, potassium, bicarbonate, phosphate, and uric acid. Urinalysis with dipstick and microscopy was performed in random urine samples. Urine protein to creatinine ratio (UPCR) was obtained from the ratio of random urine protein and creatinine [20, 21]. CKD-EPI equation was used to represent an estimated glomerular filtration rate (GFR), which was derived from GFR = 141 × min(creatinine/*κ*,1)^*α*^  × max(creatinine/*κ*,1)^−1.209^  ×  0.993^Age^  × 1.018 [if female]. In this equation, *κ* is 0.7 for females and 0.9 for males, *α* is −0.329 for females and −0.411 for males, min indicates the minimum of creatinine/*κ* or 1, and max indicates the maximum of serum creatinine/*κ* or 1 [22]. Fractional excretion of potassium (FEK) ([urine potassium × plasma creatinine]/[urine creatinine × plasma potassium] × 100), fractional excretion of phosphate (FEPO_4_) ([urine phosphate × plasma creatinine]/[urine creatinine × plasma phosphate] × 100), and fractional excretion of uric acid (FEUA) ([urine uric acid × plasma creatinine]/[urine creatinine × plasma uric acid] × 100) were calculated from serum and 24-hour urine samples [20, 21, 23]. Tubular maximal reabsorption rate of phosphate to GFR (TmPO_4_/GFR) (plasma PO_4_  − ([urine phosphate × plasma creatinine]/urine creatinine)) was calculated [18].

### 2.3. Definition and Criteria of Proximal RTD [17, 23, 24]

(i) Proteinuria = 24-hour urine protein >150 mg
(ii) Glycosuria with normoglycemia = positive glucose dipstick (or urine glucose >300 mg per day) while fasting glucose <100 mg/dL
(iii) Phosphaturia = FEPO_4_ >18% or 24-hour urine phosphate >1,200 mg
(iv) Uricosuria = FEUA >15%
(v) Renal potassium loss = hypokalemia with FEK >6.5% or 24-hour urine potassium >20 mEq per day
(vi) Renal tubular acidosis = serum bicarbonate <19 mmol/L with normal gap acidosis.

Subclinical proximal RTD was defined when 2 criteria presented, and overt proximal RTD was defined when ≥3 criteria were identified.

### 2.4. Statistical Analysis

From a previous study, we assumed a prevalence of nucleotide analogue related proximal RTD to be 15% [19]. Based on this prevalence, the required sample size was computed using OpenEpi online software to be 83 assuming a confidence limit of 7.5% and *α* level of 0.05 [25]. Eighty-three patients were required for enrollment. Data are expressed as mean ± standard deviation (SD) or median (range). Categorical variables were compared with Chi-square or Fisher's exact tests. Means and medians were analyzed by one-way analysis of variance (ANOVA) and nonparametric test, respectively. Statistical analysis was performed using SPSS version 16.0 (Chicago, IL).

## 3. Results

### 3.1. Data of CHB Patients on Nucleotide Analogue Treatment Based on the Severity of Proximal RTD (Table 1)

During the study period, 110 CHB patients who were on nucleotide analogues were screened. Fifteen patients were excluded due to the presence of significant comorbidities, for example, diabetes mellitus and hypertension. Three patients refused to participate with the study due to their personal reasons. Ninety-two CHB patients were enrolled. The mean age was 55.0 ± 10.3 years. Fifty-nine (64.1%) patients were male, with compensated cirrhosis in 28 (30.4%) patients. The mean duration of nucleotide analogue usage was 55.8 ± 29.6 (range 12–116) months. Type 2 diabetes and hypertension were present in 17 (18.5%) and 31 (33.7%) patients. The mean serum creatinine level was 0.97 ± 0.2 (range 0.49–1.50) mg/dL. The details of current antiviral drugs were as follows: ADV in 3 (3.3%) patients, TDF in 29 (31.5%) patients, ADV add-on LAM (ADV-LAM) in 11 (12.0%) patients, and TDF add-on LAM (TDF-LAM) in 49 (53.2%) patients. Among 78 patients who were on TDF regimen (TDF and TDF-LAM), 27 had been switched from ADV because of financial problems or inadequate virological response.

Subclinical and overt proximal RTD were detected in 15 (16.3%) and 9 (9.8%) patients. The mean age, the number of type 2 diabetes, and hypertension among the normal, subclinical, and overt proximal RTD groups were not different. The patients with subclinical and overt proximal RTD had longer duration of nucleotide analogue treatment (*P* = 0.01). Eleven out of 78 (14.1%) patients with TDF regimen and 4 out of 14 (28.6%) patients with ADV regimen (ADV and ADV-LAM) developed subclinical proximal RTD. Six (7.7%) patients with TDF-LAM and 3 (21.4%) patients with ADV regimen had overt proximal RTD. Thus, proximal RTD was found in 17 (21.9%) patients in the TDF regimen and 7 (50%) patients in the ADV regimen. Moreover, proximal RTD occurred in 7 out of 51 patients with TDF who never received ADV and 7 out of 13 patients with ADV who were never exposed to TDF.

Among 24 patients who were found to have proximal RTD, all but one patient who was classified as having overt proximal RTD were asymptomatic. Muscle weakness and bone pain were two complaints of this patient while severe hypophosphatemia was detected. His bone mineral density revealed osteoporosis. Bone mineral density was done in 15 patients with proximal RTD. Osteoporosis (6 patients) and osteopenia (4 patients) were found in 10 (66.7%) patients.

**Figure 1:**
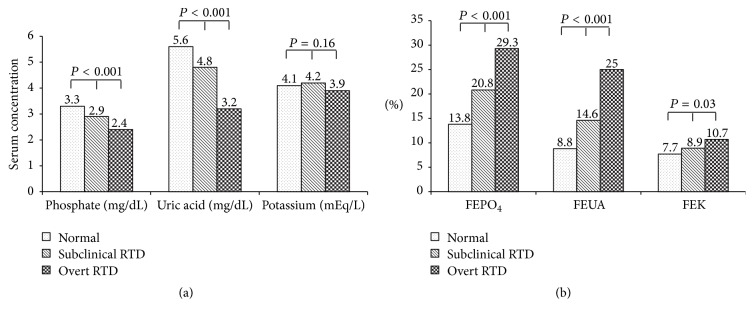
Serum phosphate, uric acid, and potassium levels among 3 groups are showed in (a). Fractional excretion of phosphate, uric acid, and potassium among 3 groups are showed in (b).* FEPO*_*4*_ *, fractional excretion of phosphate; FEUA, fractional excretion of uric acid, and FEK, fractional excretion of potassium*.

While the severity of proximal RTD worsened, urinary protein increased and renal function deteriorated progressively (Table 2). Tubular maximal reabsorption rate of phosphate to GFR (TmPO_4_/GFR) was gradually decreasing (*P* < 0.001). Serum phosphate and uric acid levels diminished in higher degree of proximal RTD (*P* < 0.001 for both) (Figure 1(a)). The lower serum phosphate and uric acid levels were explained by progressively increased renal phosphate and uric acid losses in subclinical and overt proximal RTD (*P* < 0.001 for both) as shown in Figure 1(b). Increase in urinary potassium was observed in higher degree of proximal RTD (*P* = 0.03) despite unchanged serum potassium level (*P* = 0.16). None of the patients in this study had hypokalemia from renal potassium losses. Glycosuria with normoglycemia was seen in 4 (44.4%) patients in the overt proximal RTD group. Normal gap acidosis occurred in one case in the overt proximal RTD group. Although liver enzymes were overall normal, serum alkaline phosphatase level was significantly elevated in the proximal RTD groups (Table 2).

### 3.2. The Advantage of Using the Proximal RTD Criteria to Diagnose Renal Tubular Dysfunction in Patients with Subclinical Proximal RTD in Comparison with the Recommendation of the AASLD Guideline [7, 17, 23, 24]

**Figure 2:**
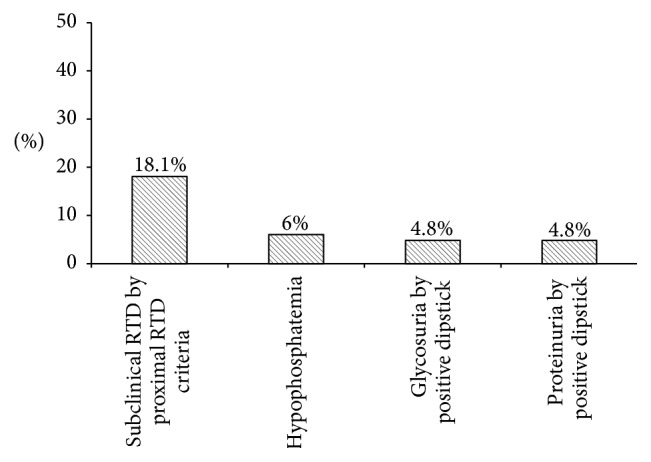
The percentage of subclinical renal tubular dysfunction defined by the proximal renal tubular dysfunction (RTD) criteria, hypophosphatemia, glycosuria, and proteinuria from positive urinary dipstick.

For the follow-up of proximal tubular function during the treatment with nucleotide analogues, the AASLD guideline suggests that serum phosphate, urine glucose, and urine protein should be performed periodically [7]. We took the opportunity to look into this issue. Out of 83 patients who did not have overt proximal RTD, 15 (18.1%) patients were found to have subclinical RTD by the proximal RTD criteria, comparing to 5 (6.0%), 4 (4.8%), and 4 (4.8%) patients who were identified to have hypophosphatemia, glycosuria by positive glucose dipstick (≥1+), and proteinuria by positive protein dipstick (≥trace), respectively (Figure 2).

### 3.3. Improvement of Proximal Renal Tubular Function at 3 Months after Discontinuation of Nucleotide Analogues (Table 3)

Nucleotide analogues were discontinued after proximal RTD was detected. In total 9 patients in the overt proximal RTD and 8 patients in the subclinical proximal RTD groups were enrolled in our on-going and follow-up study of renal tubular function after discontinuation of nucleotide analogues, and antiviral regimens were changed to ETV or LAM monotherapy. Osteoporosis was treated with bisphosphonate. Calcium carbonate and vitamin D were given to the patients with osteoporosis or osteopenia. Three months after nucleotide discontinuation, a full recovery of proximal RTD was seen in 5 (29.4%) patients. Mean GFR increased from 67.8 ± 17.0 to 74.6 ± 16.3 mL/min (*P* = 0.005) with a reduction of median 24-hour urine protein from 252 (55–939) to 88.5 (0–355) mg (*P* = 0.003). Serum phosphate and uric acid levels increased significantly at 3 months after drug discontinuation while renal loss of uric acid was significantly decreased, with a marginal decline in renal phosphate loss.

## 4. Discussion

After long-term nucleotide analogue treatment in CHB, 26% of patients developed proximal RTD in this study. Overt proximal RTD was seen in an estimated 10% of patients. The prevalence of nucleotide analogue-related proximal RTD in our study was higher than a previous report (15%), which may be due to the different criteria of proximal RTD in our study [17, 23, 24] and the other report [19]. On contrary, previous studies did not report significant reduction in renal function after long-term nucleotide analogue treatment in CHB [13–15, 26]. However, comprehensive laboratory testing for proximal renal tubular function was not performed in these reports. Forty-one (44.6%) patients currently received ADV (14 patients) or used to receive ADV prior to the switch to TDF (27 patients). Higher rates of renal impairment and Fanconi syndrome have been more reported with ADV treatment than TDF [12]. Long-term studies of CHB patients receiving TDF revealed no clinically relevant changes in renal function [13–15]. The greater number of proximal RTD in this study may be explained from the high number of ADV exposure. The issue of multiple and heterogeneous regimens of nucleotide analogue treatment in mono- and add-on therapy with LAM is one of the limitations of this study. The duration of nucleotide analogue treatment in our study (55.8 ± 29.6 months) was longer than those in previous studies (24 months and 2.4 years) [15, 26]. These results indicated that long-term nucleotide analogue treatment could cause renal damage unless the drugs had been timely withdrawn. The longstanding use of ADV and TDF can increase some risk of proximal RTD. It is worthy of note that TDF-related proximal RTD can occur in the absence of rising serum creatinine level [23, 27]. All, but one patient in this study with overt proximal RTD, presented without symptom. Thus, the presence of nucleotide analogue-related proximal RTD might not be clinically recognized unless the careful evaluation of renal tubular function was carried out. Furthermore, twenty-hour urine collection was used for the estimation of protein, phosphate, and uric acid losses instead of spot urine samples for more accurate results. From our final analysis, there was insignificant difference in the results of spot and 24-hour urine samples. Spot urine samples can be used for the monitoring of renal tubular function in clinical practice. From the AASLD guideline, serum creatinine, phosphate, urine glucose, and urine protein should be evaluated before initiation of TDF and periodically thereafter [7]. We have showed that the proximal RTD criteria performed better than the testing of serum phosphate and urine analysis with urinary dipstick to identify patients with subclinical tubular dysfunction.

In this study, phosphaturia was the most common feature when proximal RTD was described. FEPO_4_ >18% was used for the criteria of tubular phosphate loss in our study [24], while tubular reabsorption of phosphate (TRP) <82% was an alternative criterion used in some other reports [19, 23, 28]. In addition, the problem of severe tubular phosphate loss in subclinical and overt proximal RTD was confirmed with low TmPO_4_/GFR (<2.8 mg/dL) in our study. Nucleotide analogue-related Fanconi syndrome, which is an extreme degree of proximal RTD, has a particularly low incidence in CHB treatment as shown from a few case reports [29, 30]. In this study, acid loading test and the investigation of aminoaciduria were not done to confirm the diagnosis of Fanconi syndrome, although some patients with overt proximal RTD shared many features of Fanconi syndrome. Osteoporosis and osteopenia were found in 10 (66.7%) patients who had proximal RTD, which was very close to the prevalence of the reduction of bone mineral density in patients who were treated with nucleotide analogues in a previous report [31]. Long-term hypophosphatemia with decreased renal phosphate absorption can lead to osteomalacia and impaired bone health [29].

In long-term ADV usage in CHB treatment, age and the duration of ADV usage were the predictors of the development of proximal RTD [17]. The other study revealed that preexisting renal dysfunction was the predisposing factor of nucleotide analogue-related proximal RTD in patients with CHB [19]. From our study, the duration of nucleotide analogue was the only significant factor associated with proximal RTD. Age was not found to be a risk factor, which may be due to the lower number of older patients in this study. TDF is eliminated unchanged in urine by glomerular filtration and proximal tubular secretion. About 20–30% of TDF is absorbed at proximal tubule cells through basal membrane organic anion transporter (hOAT1 and hOAT3) and exits across apical membrane via multidrug resistant-associated protein 4 (MRP4) and MRP2 [27]. Polymorphism of genes (ABCC4 and ABCC2) encoding these transporters can result in TDF-related proximal RTD [27]. Hence, proximal RTD can occur in an individual, but not in others who are treated with the same nucleotide analogue [27].

Once proximal RTD occurs, TDF should be discontinued and replaced with other antiviral drugs with consideration for the previous history of HBV drug resistance [7]. The proximal renal tubular function needs to be followed up closely. If there is recovery of proximal RTD after discontinuation of nucleotide analogues, it will help to confirm that the proximal RTD is related to long-term nucleotide analogue treatment [10]. A full recovery of renal tubular function was seen in about 29% at three months after discontinuation of nucleotide analogues in this study. The result of long-term outcome of discontinuation of nucleotide analogues still remains to be investigated. Therefore, early detection of nucleotide analogue-related proximal RTD and early withdrawal of the drug are the fundamental approaches to avoid irreversible proximal renal tubular injury [10]. The role of new nucleotide analogues without nephrotoxicity such as tenofovir alafenamide (TAF) in the prevention and reduction of nucleotide analogue-related nephrotoxicity is under investigation [32].

There are some limitations of our study that need to be addressed. It was a single center and cross-sectional study which lacked the baseline data of patients before the initiation of nucleotide analogues. As a tertiary center, patients with more severe liver diseases and multiple medical conditions were enrolled to the study. During the course of treatment, nucleotide analogue was switched in some patients due to problems such as financial issues and inadequate virological response.

## 5. Conclusion

Our study has demonstrated that proximal RTD and a decline in GFR are associated with long-term nucleotide analogue treatment in CHB patients. Comprehensive laboratory testing is the crucial step for the early detection of proximal RTD to avoid irreversible renal dysfunction. Genetic studies in the polymorphisms of drug transporters should be investigated to determine their roles in this condition. Further well-designed multicenter studies should be carried out on a larger scale basis.

## Figures and Tables

**Table 1:** Demographics of 92 patients on nucleotide analogues according to the severity of proximal renal tubular dysfunction (RTD).

	Normal (*n* = 68)	Subclinical proximal RTD (*n* = 15)	Overt proximal RTD (*n* = 9)	*P*
Age (years)	54.0 ± 8.8	56.2 ± 12.5	60.2 ± 15.5	0.21
Male, *n* (%)	43 (63.2)	11 (73.3)	5 (55.6)	0.65
Liver cirrhosis, *n* (%)	21 (30.9)	4 (26.7)	3 (33.3)	0.93
Type 2 diabetes, *n* (%)	12 (17.6)	3 (20.0)	2 (22.2%)	0.93
Hypertension, *n* (%)	20 (29.4)	8 (53.3)	3 (33.3)	0.21
Duration of nucleotide analogue taking (months)	50.7 ± 28.9	65.1 ± 30.2	78.7 ± 20.2	0.01

Data are expressed as mean ± SD unless otherwise indicated.

**Table 2:** Blood and urine chemistries of 92 patients treated with nucleotide analogues according to the severity of proximal renal tubular dysfunction (RTD).

	Normal (*n* = 68)	Subclinical proximal RTD (*n* = 15)	Overt proximal RTD (*n* = 9)	*P*
AST (IU/L)	38.9 ± 21.7	34.4 ± 9.3	29.1 ± 7.6	0.30
ALT (IU/L)	53.8 ± 35.8	50.7 ± 26.9	35.7 ± 7.7	0.31
ALP (IU/L)	74.7 ± 18.8	89.3 ± 28.4	107.9 ± 60.3	0.036
Creatinine (mg/dL)	0.92 ± 0.2	1.05 ± 0.2	1.19 ± 0.2	0.002
GFR by CKD-EPI (mL/min)	86.7 ± 16.6	74.5 ± 16.0	59.9 ± 13.6	<0.001
Proteinuria, *n* (%)	9 (13.2)	10 (66.7)	9 (100)	<0.001
Phosphaturia, *n* (%)	9 (13.2)	13 (86.7)	9 (100)	<0.001
Uricosuria, *n* (%)	0	5 (33.3)	9 (100)	<0.001
Glycosuria with normoglycemia, *n* (%)	2 (2.9)	2 (13.3)	4 (44.4)	<0.001
Renal potassium loss, *n* (%)	0	0	0	
Normal gap acidosis, *n* (%)	0	0	1 (11.1)	0.009
Median 24-hour urine protein (mg)	86 (0–425)	158 (55–437)	408 (190–939)	<0.001
Median UPCR (mg/mg)	0.10 (0–0.49)	0.18 (0.08–0.42)	0.54 (0.29–1.14)	<0.001
TmPO_4_/GFR (mg/dL)	2.9 ± 0.5	2.3 ± 0.3	1.7 ± 0.7	<0.001

AST, aspartate aminotransferase; ALT, alanine aminotransferase; ALP, alkaline phosphatase; GFR, glomerular filtration rate; UPCR, urine protein to creatinine ratio; TmPO_4_/GFR, tubular maximal reabsorption rate of phosphate to GFR.

Data are expressed as mean ± SD unless otherwise indicated.

**Table 3:** Blood and urine chemistries at baseline and 3 months after discontinuation of nucleotide analogues in 17 patients with proximal renal tubular dysfunction (RTD).

	At baseline	Three months after drug discontinuation	*P*
Creatinine (mg/dL)	1.04 ± 0.2	0.96 ± 0.2	0.005
GFR by CKD-EPI (mL/min)	67.8 ± 17.0	74.6 ± 16.3	0.005
Serum phosphate (mg/dL)	2.5 ± 0.6	3.1 ± 0.6	0.002
Serum uric acid (mg/dL)	3.4 ± 1.1	3.9 ± 1.0	<0.001
Serum potassium (mEq/L)	4.0 ± 0.3	4.0 ± 0.3	0.53
FEPO_4_ (%)	25.5 ± 9.0	22.5 ± 12.1	0.13
FEUA (%)	22.9 ± 9.2	18.1 ± 8.3	0.003
FEK (%)	9.8 ± 4.7	13.7 ± 14.7	0.40
Median 24-hour urine protein (mg)	252 (55–939)	88.5 (0–355)	0.003

GFR, glomerular filtration rate; FEPO_4_, fractional excretion of phosphate; FEUA, fractional excretion of uric acid; FEK, fractional excretion of potassium.

Data are expressed as mean ± SD unless otherwise indicated.

## Abbreviations

CHB: Chronic hepatitis B
RTD: Renal tubular dysfunction
LAM: Lamivudine
FEPO_4_: Fractional excretion of phosphate
FEUA: Fractional excretion of uric acid
FEK: Fractional excretion of potassium
HBV: Hepatitis B virus
TDF: Tenofovir disoproxil fumarate
ADV: Adefovir dipivoxil
UPCR: Urine protein to creatinine ratio
GFR: Glomerular filtration rate
TAF: Tenofovir alafenamide.
